# Supplementary material for: Advancing wavefront shaping with resonant nonlocal metasurfaces: beyond the limitations of lookup tables
Source: Sci Rep. 2024 Jan 18;14:1555. doi: 10.1038/s41598-024-51898-8 (PMC10796368; doi:10.1038/s41598-024-51898-8)
Supplement: Supplementary file 1 — Supplementary Information. [file 41598_2024_51898_MOESM1_ESM.pdf]

Supplementary information

Enzo Isnard[1,2], Sébastien Héron[2], Stéphane Lanteri[1], and Mahmoud Elsawy[1,*]

[1]Université Côte d'Azur, Inria, CNRS, LJAD, 06902 Sophia Antipolis Cedex, France
[2]THALES Research & Technology, 1 avenue Augustin Fresnel, 91120 Palaiseau, France
[*] mahmoud.elsawy@inria.fr

1.1 Geometric parameters of the beam deflectors in transmission

Parameters	Local design	Optimized design
r_1	205.44 nm	210.20 nm
r_2	194.24 nm	197.13 nm
r_3	188.13 nm	186.35 nm
r_4	183.26 nm	180.82 nm
r_5	178.38 nm	176.58 nm
r_6	172.66 nm	173.62 nm
r_7	161.63 nm	161.48 nm
r_8	130.00 nm	262.87 nm
dx_1	620.00 nm	603.51 nm
dx_2	620.00 nm	603.12 nm
dx_3	620.00 nm	601.74 nm
dx_4	620.00 nm	599.24 nm
dx_5	620.00 nm	605.30 nm
dx_6	620.00 nm	607.26 nm
dx_7	620.00 nm	604.93 nm

Supplementary Table 1. Geometric parameters of the beam deflector designs presented in section 2.1.

1.2 Geometric parameters of the EDOF metalenses

Parameter	Parabolic	Log-asphere	Optimized fixed h	Optimized varying h
r_1	177.73 nm	156.39 nm	144.40 nm	130.00 nm
r_2	192.07 nm	174.19 nm	181.00 nm	155.95 nm
r_3	139.56 nm	182.23 nm	178.93 nm	171.93 nm
r_4	174.94 nm	189.93 nm	184.89 nm	167.24 nm
r_5	184.24 nm	203.07 nm	188.38 nm	179.67 nm
r_6	192.11 nm	142.56 nm	197.54 nm	184.94 nm
r_7	202.64 nm	168.07 nm	201.83 nm	189.14 nm
r_8	218.37 nm	175.16 nm	209.44 nm	195.77 nm
r_9	232.12 nm	177.87 nm	138.84 nm	202.92 nm
dx_0	342.67 nm	342.67 nm	318.80 nm	306.93 nm
dx_1	688.89 nm	688.89 nm	630.13 nm	600.90 nm
dx_2	688.89 nm	688.89 nm	634.97 nm	693.27 nm
dx_3	688.89 nm	688.89 nm	655.32 nm	647.38 nm
dx_4	688.89 nm	688.89 nm	643.28 nm	621.93 nm
dx_5	688.89 nm	688.89 nm	672.79 nm	510.00 nm
dx_6	688.89 nm	688.89 nm	612.42 nm	510.00 nm
dx_7	688.89 nm	688.89 nm	670.47 nm	558.42 nm
dx_8	688.89 nm	688.89 nm	614.86 nm	693.27 nm
h	170.00 nm	170.00 nm	170.00 nm	189.49 nm

Supplementary Table 2. Geometric parameters of the EDOF metalens designs presented in section 2.2.

1.3 Geometric parameters of the beam deflectors in reflection

Parameter	Local design	Nonlocal fixed H and D	Nonlocal varying H and D
W_1	155.00 nm	168 nm	175 nm
W_2	166.24 nm	234 nm	252.5 nm
W_3	220.00 nm	107 nm	107 nm
H	400 nm	400 nm	405 nm
D	140 nm	140 nm	110 nm
L_1	139 nm	106 nm	77 nm
L_2	107 nm	50 nm	93 nm

Supplementary Table 3. Optimized parameters for the 3 element case depicted in Fig.3(b). The corresponding deflection efficiency together with the field profile for each case are depicted in Figs.3(c) and (d), respectively.

Parameter	Local design	Nonlocal fixed H and D	Nonlocal varying H and D
W_1	154.90 nm	239 nm	128 nm
W_2	156.73 nm	105.6 nm	145 nm
W_3	172.98 nm	110 nm	199 nm
W_4	213.96 nm	152 nm	96.55 nm
H	400 nm	400 nm	350.45 nm
D	140 nm	140 nm	250 nm
L_1	144 nm	106 nm	84.36 nm
L_2	135 nm	120 nm	108 nm
L_3	106.5 nm	74.8 nm	98.54 nm

Supplementary Table 4. Optimized parameters for the 4 element case depicted in Sup. Fig.3.

1.4 Supplementary Figures

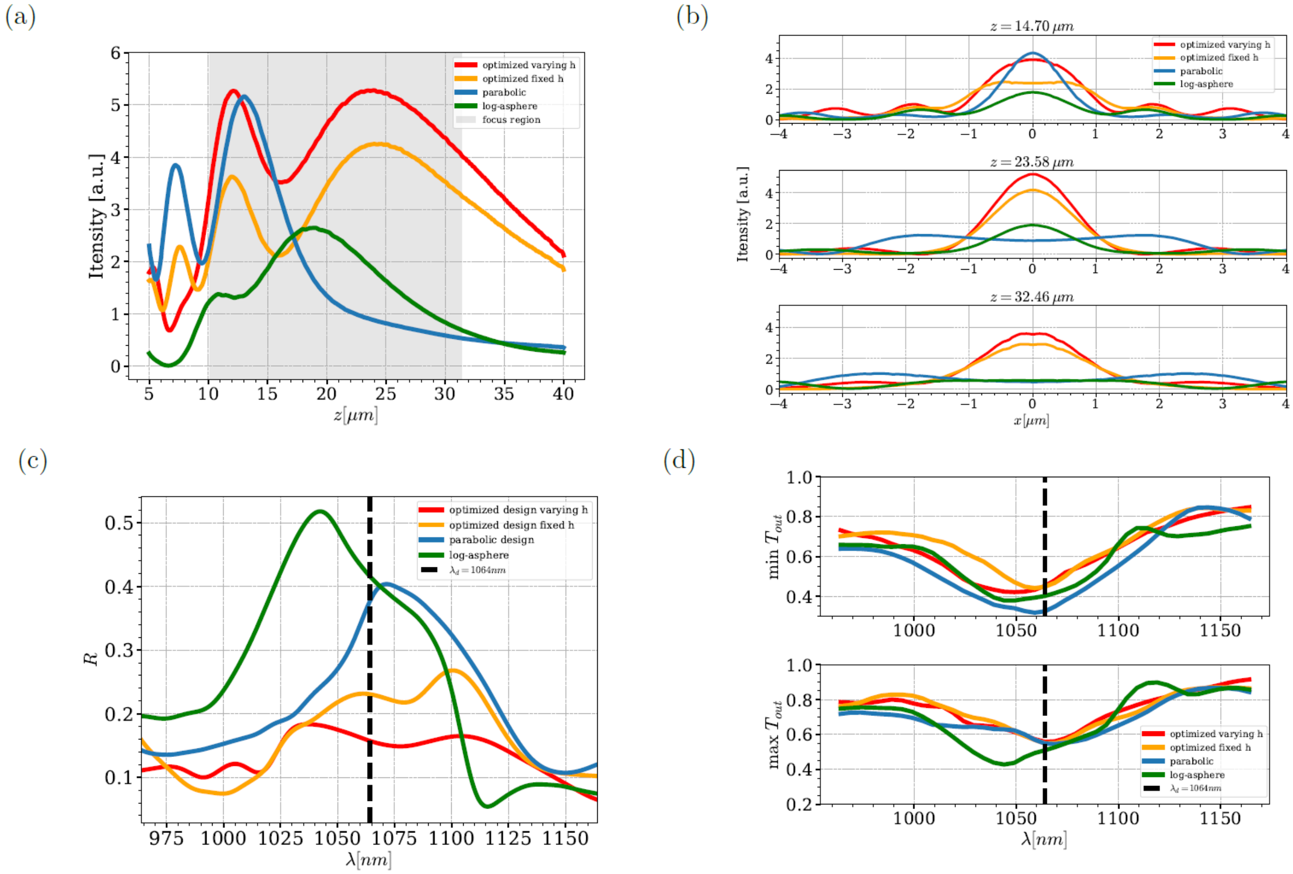

Supplementary Figure 1. Optimization results for the EDOF metalens.(a) Intensity of the lens along the optical axis. The gray region represents the region in which we extend the depth of focus. The optimized with a varying h outperforms clearly the parabolic design. The one with a fixed h has a intensity profile similar to the one with varying h but with consistently less intensity. (b) PSFs at 1064nm of the different designs at f, f+2.5 DOF and f+5 DOF. Optimized design PSFs keep a consistent shape at the three locations while the classical designs ones have flattened considerably at f + 2.5 DOF and f + 5 DOF. (c) Reflection of each design. The optimized designs have considerably less back-scattering than the classical ones. Introducing h as an optimization variable enables to reduce the reflection by 8%. (d) Plot of the minimum and maximum of the part of the incident power which is scattered forward but that doesn't hit the focus surface over 20 equally spaced rectangles in the target region. It represents the large part of loose of focus efficiency. It is mainly due to the small aperture of the lens ($\sim 10\lambda$) that causes inevitable diffraction.

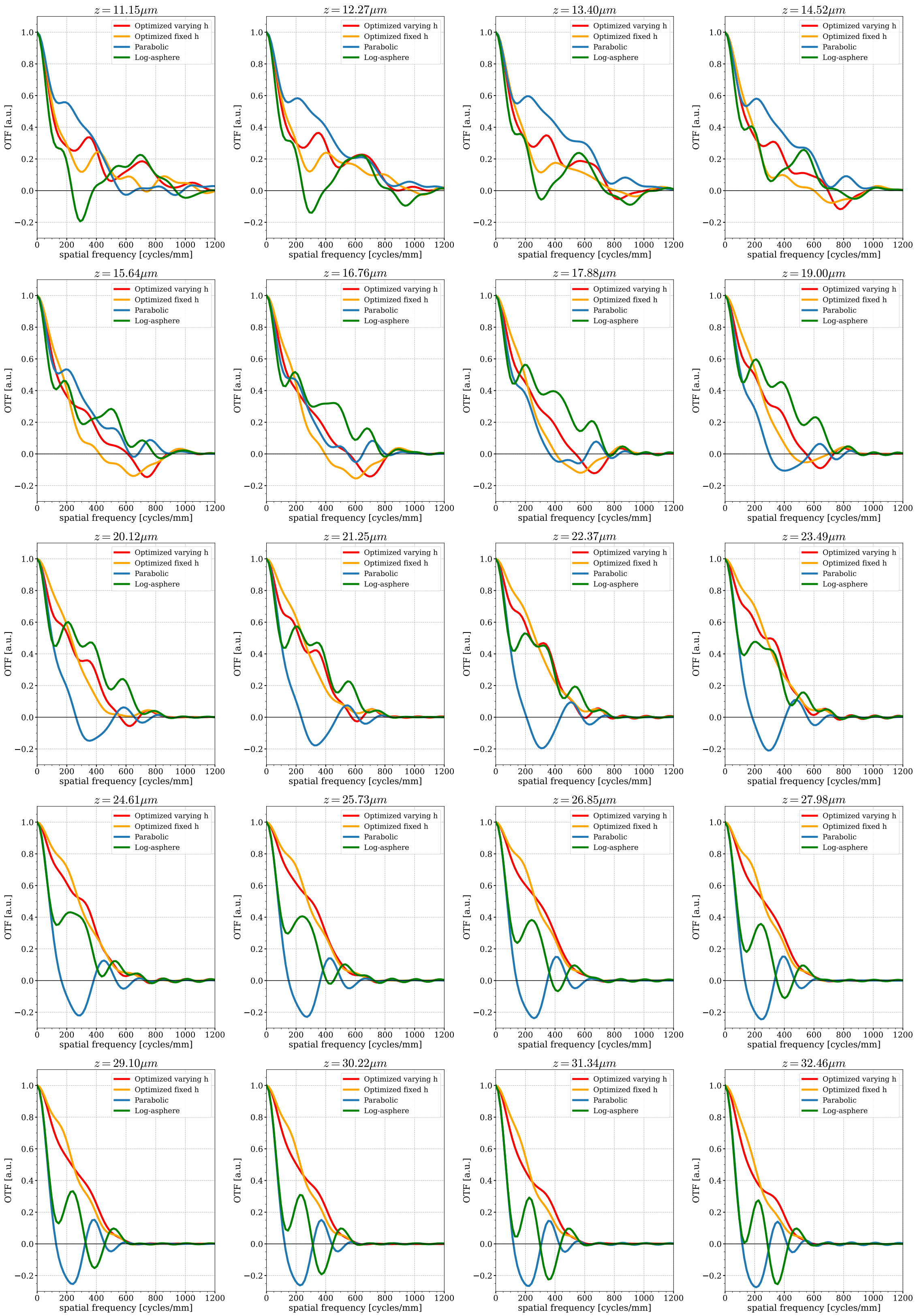

Supplementary Figure 2. OTFs at 20 different focal planes equally spaced from f − DOF to f + 5 DOF. Optimized designs exhibit a more consistent optical resolution than the classical ones.

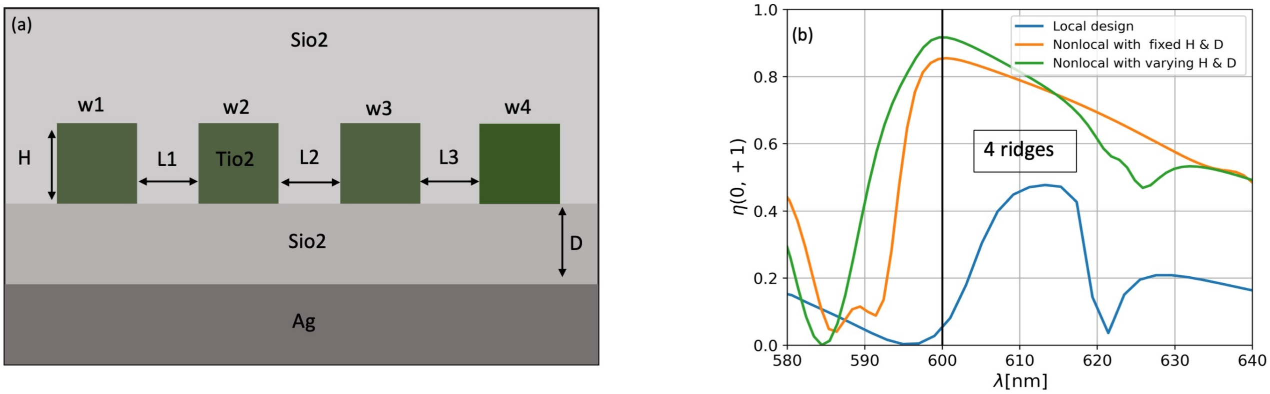

Supplementary Figure 3. Optimization results for the 4 element case in reflection. (a) Geometry under consideration. (b) Deflection performance comparison between various scenarios similar to the 3 element case given in Fig.2. The optimization parameters are given in Tab. 4.
